# Early identification and treatment of women's cardiovascular risk factors prevents cardiovascular disease, saves lives, and protects future generations: Policy recommendations and take action plan utilizing policy levers

**DOI:** 10.1002/clc.23921

**Published:** 2022-09-20

**Authors:** Susan L. Ivey, Hattie Rees Hanley, Catrina Taylor, Eveline Stock, Nirali Vora, Jenny Woo, Sara Johnson, C. Noel Bairey Merz, Right Care Women's Cardiovascular Writing Group

**Affiliations:** ^1^ UC Berkeley, School of Public Health Berkeley California USA; ^2^ California Department of Public Health Sacramento California USA; ^3^ UCSF, School of Medicine, Cardiology San Francisco California USA; ^4^ School of Medicine, Neurology Stanford University Stanford California USA; ^5^ Obstetrics and Gynecology Alta Bates Summit Medical Center Berkeley California USA; ^6^ Barbra Streisand Women's Heart Center, Cedars‐Sinai Medical Center Smidt Heart Institute Los Angeles California USA

**Keywords:** cardiovascular, women

## Abstract

Cardiovascular diseases (CVD) including heart attacks, strokes, heart failure, and uncontrolled hypertension are leading causes of death among women of all ages. Despite efforts to increase awareness about CVD among women, over the past decade there has been stagnation in the reduction of CVD in women, and CVD among younger women and women of color has in fact increased. We recommend taking action using policy levers to address CVD in women including: (1) Promoting periodic screening for risk factors including blood pressure, lipids/cholesterol, diabetes for all women starting at 18–21 years, with calculated atherosclerotic CVD (ASCVD) risk score use among women 40 years or older. (2) Considering coronary artery calcium (CAC) screening for those with intermediate risk per current guidelines. (3) Enhancing Obstetrics and Gynecology and primary care physician education on reproductive age CVD risk markers, and that follow‐up is needed, including extended postpartum follow‐up. (4) Offering Health Coaching/motivational Interviewing to support behavior change. (5) Funding demonstration projects using different care models. (6) Creating a Stop High Blood Pressure consult line (for providers and patients) and providing other support resources with actions consumers can take, modeled after the California tobacco quit line. And (7) Requiring inclusion of adverse pregnancy outcomes in all Electronic Health Records, with reminder systems to follow‐up on hypertension post‐partum.

## 1 INTRODUCTION

Cardiovascular diseases (CVD) including heart attacks, strokes, heart failure, and uncontrolled hypertension are leading causes of death among women of all ages.^1^ These conditions strike women at younger ages than is commonly understood, causing death, disability, and family devastation. Despite efforts to increase awareness about CVD among women, over the past decade there has been stagnation in the reduction of CVD in women, and CVD among younger women and women of color has in fact increased.^2^ Many heart attacks, strokes, and hypertensive conditions are preventable with early detection and awareness of risk factors. Lifestyle changes and better application of guideline‐based care can prevent premature deaths from CVD.^3^ Conversely, women's retention of adverse lifestyles, along with provider underestimation of women's risk categorization and failure to identify and treat CV risks and clinical disease, can widen gender disparities in CVD prevention.^4^

There is a misperception that CVD is not an issue for women until after menopause. The data speak otherwise. In 2019, the total CVD (e.g., hypertensive, ischemic heart, and cerebrovascular disease) crude death rates per 100 000 US women occurred at higher rates than breast and cervical cancer, combined, from age 35+ onward (Table 1)^5^. Women are more likely to die from CVD than from any other condition, including those women aged 35–44, 45–54, and 55–64 years of age.^5^ Additionally, young women ages 18–35 have 44% more ischemic stroke than men,^6^ and stroke is a leading cause of death among Black women.^7^ CVD also accounts for about 33% of the rising maternal deaths. Thus, young women are especially important to target for screening, treatment, and research because CVD prevention during this life stage can have great personal and societal health benefits.^8^

**Table 1:** Age‐stratified crude death rates per 100 000 Women—US (2019)

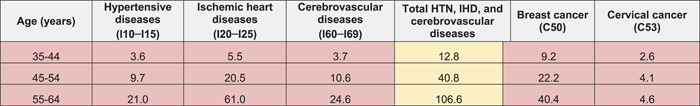

*Note*: Centers for Disease Control and Prevention, National Center for Health Statistics. Underlying cause of death 1999–2019 on CDC WONDER Online Database, released in 2020. Data are from the Multiple Cause of Death Files, 1999–2019, as compiled from data provided by the 57 vital statistics jurisdictions through the Vital Statistics Cooperative Program. Accessed at http://wonder.cdc.gov/ucd-icd10.html on Oct 25, 2021. Reprinted with permission.^5^

## 2 CARDIOVASCULAR RISK FACTORS IN WOMEN

**Figure 1:**
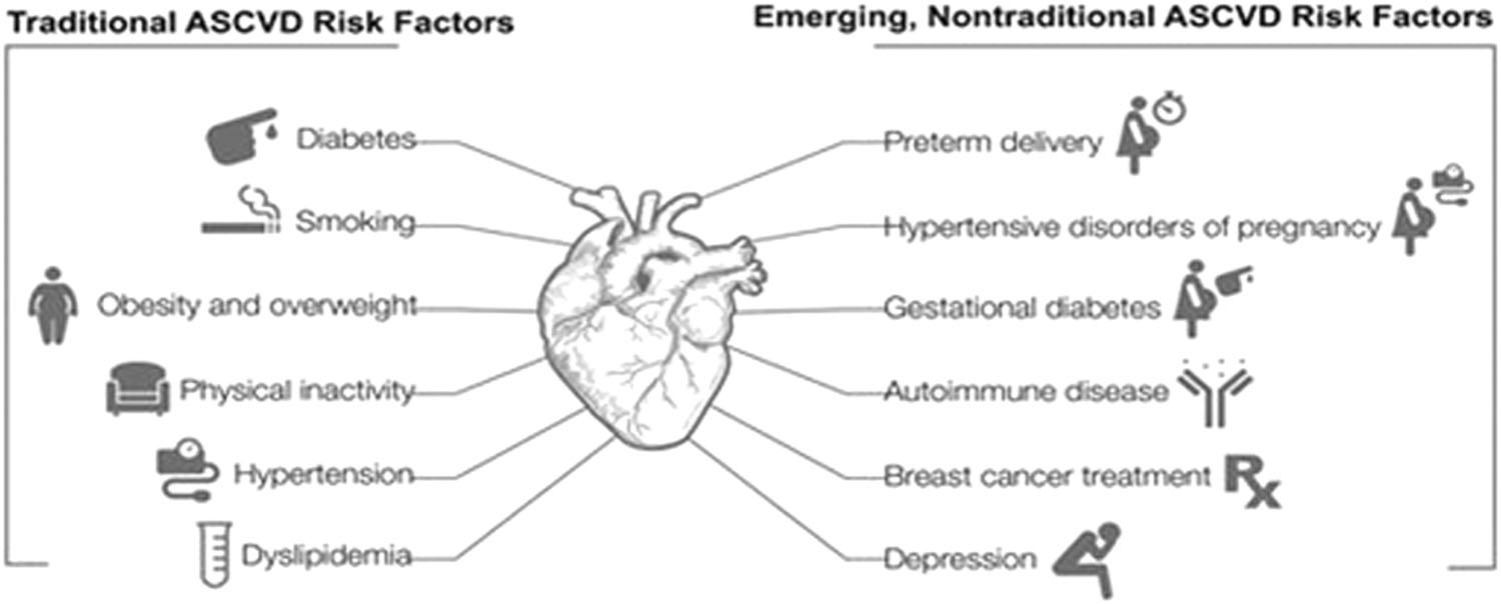
Atherosclerotic risk factors in women reprinted with permission.^2^ ASCVD, atherosclerotic cardiovascular diseases.

While women have many traditional cardiovascular risk factors including dyslipidemia, some risks—including smoking and diabetes—have a greater impact on coronary heart disease risk in women compared with men.^9^ Women may also be at risk for heart attack and stroke based on adverse pregnancy outcomes, hormonal factors which change over time, and increased rates of autoimmune conditions (lupus/rheumatoid arthritis)^10^, ^11^ (Figure 1).^2^ Women also have high rates of depression and other conditions that impact proinflammatory factors, thus increasing their vulnerability to CVD. Moreover, being African American, being pregnant at older ages (>35 years), or having obesity boost maternal risk.^12^ CVD risk factors can also adversely impact the fetus and contribute to future CVD risk in the next generation.^13^ With a trend towards pregnancies later in life, more attention is needed to identify and manage women's CVD risk in the preconception period.

**Figure 2:**
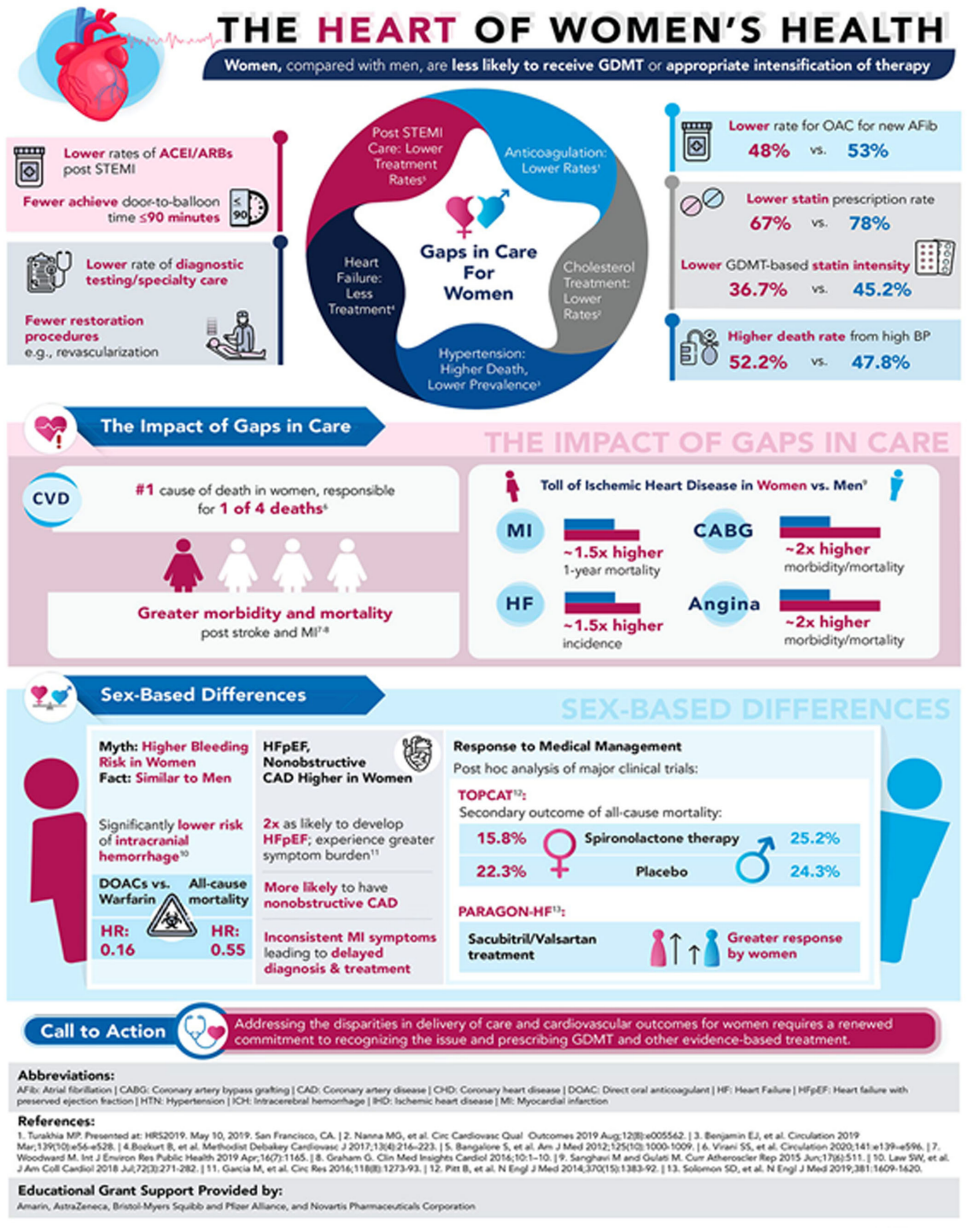
The heart of women's health reprinted with permission^15^

Despite these known risks, young women are less likely to be screened, be provided with early preventive care, achieve targets for cholesterol or Hemoglobin A1c (HbA1c), reach target blood pressure goals, and are less aggressively treated with medical therapies and percutaneous or surgical interventions despite studies showing benefit in women as well as men^4^, ^14^ (Figure 2).^15^ Despite similarities in medication exposure, women are less likely than men to achieve blood pressure (BP), low density lipoprotein‐cholesterol, and HbA1c targets after a coronary event, highlighting the importance of achieving risk factor control earlier in life.^16^ Another factor to be considered is that, in many situations, pharmacological treatment is begun too late, when nonreversible atherosclerotic plaque is already established. This is one justification for primordial (lifestyle and diet) and primary prevention with an aim of ASCVD risk mitigation. Pharmacologic management of BP, diabetes, and dyslipidemia is safe and effective in at‐risk young women.^2^ The “black box” warning for statins in pregnancy has also been removed due to evidence of safety.^17^

Early CV risk factor analysis and diagnostics can identify risks and predict CVD in young women. The updated 2020 Recommendations for the Updated Primary Prevention of CVD in Women^18^ considers the presence of polycystic ovary syndrome, premature menopause, and pregnancy‐associated conditions like pre‐eclampsia, gestational diabetes mellitus, gestational hypertension, or systemic autoimmune collagen‐vascular disease (e.g., lupus or rheumatoid arthritis) as risk‐enhancing factors for future CVD. The joint 2018 American Heart Association (AHA) and American College of Obstetrics and Gynecology (Ob‐Gyn) Presidential Advisory endorses having Ob‐Gyn providers screen for CVD and cardiovascular risk factors in women by eliciting a full patient history to reveal critical clues about patient risk factors and trigger appropriate referrals.^19^ Preventive care decision‐making also requires providers to have and use unbiased tools to assign women to the correct risk category so women can receive guideline‐based care and recommended lifestyle changes, regardless of perceived health. Physicians targeting risk factors for treatment can reduce heart attacks and strokes.

A recent survey of US cardiologists, CV team members, and trainees showed that although CVD is the number‐one cause of pregnancy‐related deaths in the United States, there are significant gaps in knowledge and confidence among providers pertaining to care of pregnant and postpartum women with CVD. This finding supports the need for developing new standards for training and educating members of the cardiology and obstetrics workforce to optimize the care provided to young mothers, and to facilitate the expansion of dedicated cardio‐obstetrics centers.^20^ These efforts will improve the care we provide to women at risk for CVD who are planning or experiencing pregnancy and may help reverse the alarming increase in rates of maternal morbidity and mortality experienced in the United States.

## 3 RISK FACTOR INTERVENTIONS TO REDUCE CARDIOVASCULAR RISK IN WOMEN

The AHA constructed their 2020 Impact Goal to improve the CV health of all Americans with seven health metrics defining ideal CV health.^21^ Based on four behavioral factors (smoking, physical activity, diet, and weight) and three health factors (total cholesterol, blood pressure, and metabolic‐blood sugar control), scores are divided into ideal, intermediate, or poor levels of attainment. Providing feedback to patients about their risk level and tracking their progress can be a simple way to improve behaviors. Preventive care includes empowering women to know their blood pressure and cholesterol numbers, supporting women in achieving healthy lifestyle (nonsmoking, physically active, eating plant‐centric Mediterranean diets), and understanding the many social determinants of health that can impact adoption of a healthy lifestyle.^22^ Motivational interviewing and team‐based care can support a variety of behavior changes, with evidence strongest for smoking cessation, physical activity changes, and behavior changes among persons with metabolic syndrome.^23^

**Figure 3:**
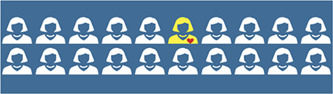
Subclinical coronary atherosclerosis in women adapted from^5^

The NIH‐funded CARDIA study of atherosclerosis among the young (conducted at Kaiser Oakland research clinics and other national centers since 1985) found that by age 45, ∼1 in 20 women already tested positive for calcium deposits in their coronary arteries, concrete evidence of established atherosclerosis and that biological mechanisms leading to heart attack and stroke are already in motion, likely to progress if left untreated (Figure 3).^5^ This level of subclinical atherosclerosis among women is well known based on information from little‐used heart computerized tomography (CT) scans with a low category of radiation exposure, similar to the category of mammography. However, while heart CT findings that are positive for calcium in the coronary arteries indicate higher risk for heart attacks and strokes, optimal lifestyle and medication management can reduce this risk. Finding these vulnerable women and prioritizing them for preventive therapies is key.

**Figure 4:**
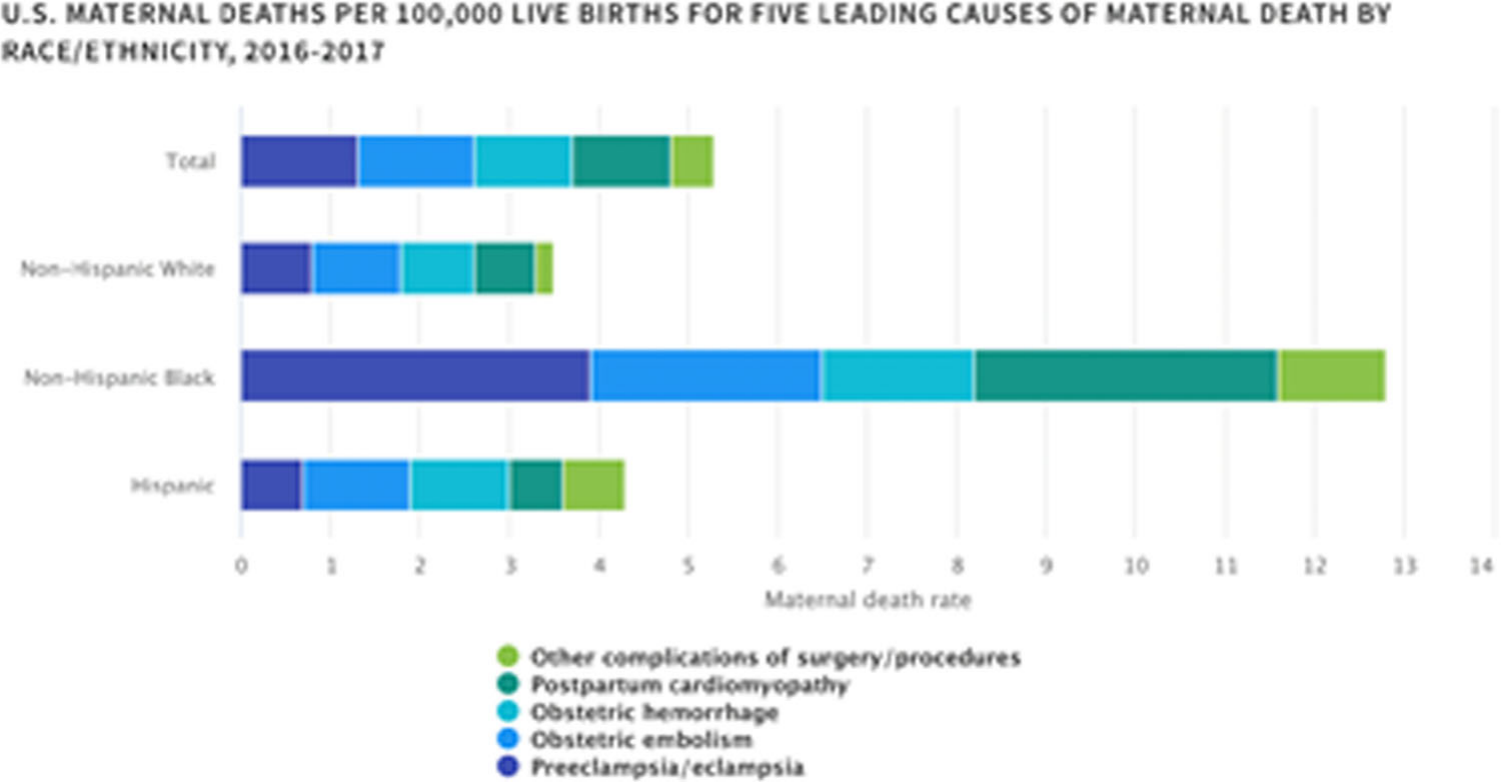
Black women over three times more likely to die in pregnancy, postpartum reprinted with permission^26^

A woman's reproductive life stage offers a unique opportunity to identify and reduce CV risk, given 86% of women will have a pregnancy.^24^ Adverse pregnancy outcomes (e.g., hypertensive disorders of pregnancy, small‐for‐gestational‐age birth, preterm birth, and stillbirth) are prevalent in more than one in five women,^25^ are most common in non‐Hispanic Black women (Figure 4)^26^ and are associated independently with CV risk, indicating that adverse pregnancy outcomes require careful monitoring.^27^ Between 2011 and 2019, rates of hypertensive disorders of pregnancy in individuals with a singleton first live birth increased in the United States across all race and ethnicity subgroups, however the rates were noted to be especially high among Filipinas (among all non‐Hispanic Asian subgroups in 2019) (92.5 [95% confidence interval (CI), 86.3–98.8] per 1000 live births), and among Puerto Rican women (among other Hispanic/Latina subgroups in 2019) (98.6 [95% CI, 94.2–102.9] per 1000 live births).^28^ The need for risk assessment and follow‐up before, during, and after pregnancy^29^, ^30^ is evident in multiple guidelines, including those from AHA.

Pregnancy is a time of both increased healthcare usage as well as increased motivation,^31^ which can be leveraged to support lifestyle changes such as exercise^32^ and Mediterranean diet^33^ that improve both pregnancy outcomes and lifelong cardiovascular health. Many women will be motivated by the prospect of improving outcomes for their baby, highlighting the importance of informing women that supporting their own prenatal health will confer benefits to their child after birth. Efforts to address pregnancy risks, such as identifying and treating gestational diabetes, and prescribing prenatal aspirin^34^ for pre‐eclampsia prevention, can be accompanied by education about associated future CV risks and the need to continue to assess and mitigate those risks following pregnancy. In the postpartum period, programs such as transition clinics and group visits, as well as telehealth interventions, are being developed that can help maintain care linkages and lifestyle changes so that ongoing CV risk can be addressed.^35^

Peripartum depression is a comorbidity in many adverse pregnancy outcomes, and represents a barrier to ongoing engagement with care, yet only a minority of women (18%) seek treatment for perinatal depression.^36^ Many women may not engage with mental healthcare nor want to take antidepressant medications.^37^ A range of options to improve mental health, including mindfulness^38^ and sleep interventions,^39^ may be useful adjuncts and can be part of an anti‐inflammatory lifestyle that improves cardiovascular risks. Increased provider awareness of cardiovascular risks conferred by adverse pregnancy outcomes, treatment of comorbid postpartum depression, and awareness of other challenges to patient engagement such as demands of new parenting, as well as studies of interventions that help Black, Brown, and Indigenous women, will be essential.

## 4 POLICY RECOMMENDATIONS

**Figure 5:**
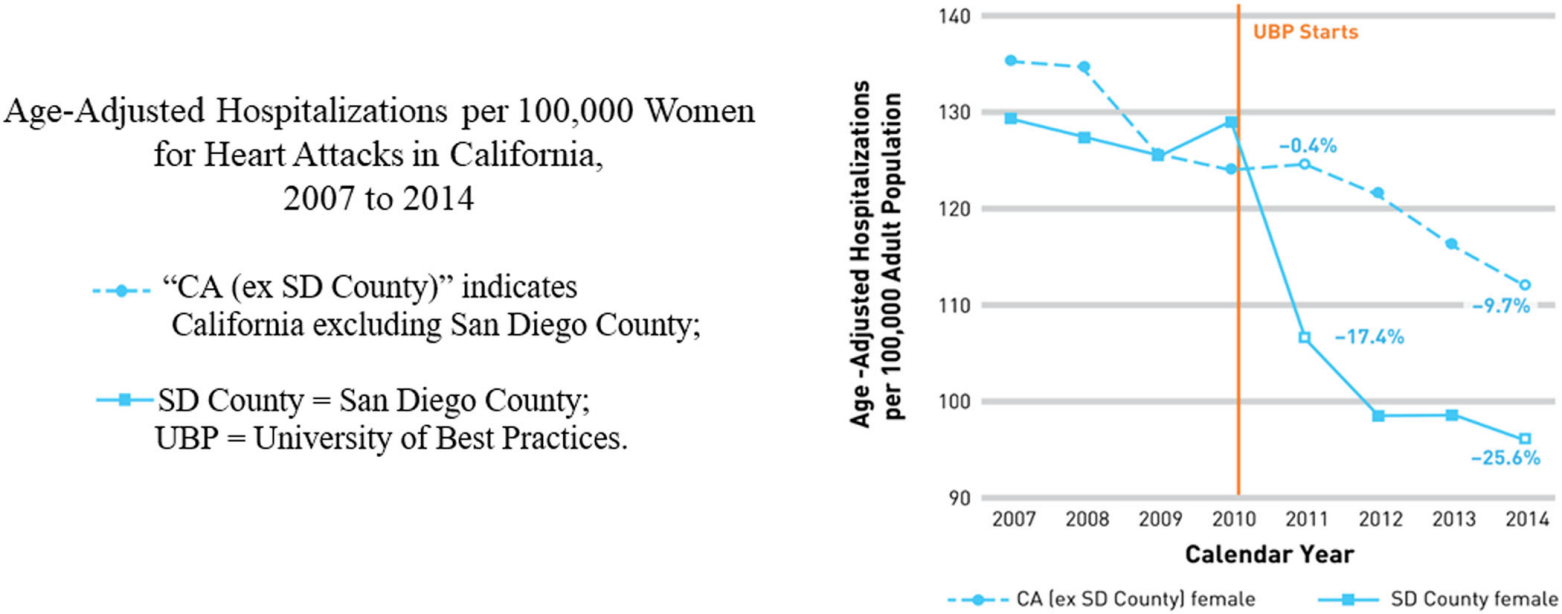
Right Care Initiative demonstration project reduces heart attacks in women adapted from^40^

Given these findings and evidence, the following policy recommendations and take action items are recommended to improve heart health in women:

1) Consistently implement current guidelines for CVD risk factor screening and treatment in women. For example, the Right Care Initiative's NIH‐funded San Diego demonstration project to drive guideline‐based care was associated with rapid reductions in heart attack hospitalizations for women (Figure 5).^40^
2) Increase identification of very young women with familial hypercholesteremia and early onset hypertension.
3) Improve uptake of physician/mid‐level provider CVD risk factor screening and treatment during women's reproductive age period, including screening for adverse pregnancy outcomes.
4) Expand postpartum Medicaid coverage in every state for at least 12 months to allow for risk assessment and treatment postdelivery.

## 5 TAKE ACTION UTILIZING POLICY LEVERS

1. Promote periodic screening for risk factors including blood pressure, lipids/cholesterol, diabetes for all women starting at 18–21 years, with calculated ASCVD risk score use among women 40 years or older. Advocate for improved risk scoring models for women.
2. Consider coronary artery calcium screening for those with intermediate risk per current guidelines.^41^ For those with family history of early heart attacks and strokes, offer the heart CT when the patient is 10 years younger than when an immediate family member suffered a heart attack or stroke.
3. Enhance Ob‐Gyn and primary care education on reproductive age CVD risk markers, and that follow‐up is needed, including extended postpartum follow‐up.
4. Offer Coaching/Motivational Interviewing to support behavior change. Teach pregnant women to monitor BP at home. Teach young women to use self‐measured blood pressure monitoring in child‐bearing years: “Heart Healthy Mamas for Healthy Babies.”
5. Fund demonstration projects using different care models: for instance, NPs embedded in Ob‐Gyn practices to screen for CVD and risks; prepartum and postpartum health coaching; consistent referral to Primary Care (Family Medicine, Internal Medicine, nurse practitioners); and team‐based care models that include pharmacist protocol management of high blood pressure and lipids.
6. Create a Stop High Blood Pressure consult line (for providers and patients) and provide other support resources with actions consumers can take, modeled after the California tobacco quit line.
7. Require inclusion of adverse pregnancy outcomes in the Electronic Health Record with reminder systems to follow‐up on hypertension postpartum (e.g., LADDR UC EHR registry, or other registries).

## CONFLICT OF INTEREST

Dr. C. Noel Bairey Merz, serves as Board of Director for iRhythm, fees paid through CSMC from Abbott Diagnostics and Sanofi. The remaining authors declare no conflict of interest.

## Data Availability

Data are available on request from the authors.
